# Research progress of Ustekinumab in the treatment of inflammatory bowel disease

**DOI:** 10.3389/fimmu.2024.1322054

**Published:** 2024-02-22

**Authors:** Weilin Zhang, Guoqiang Zhong, Xingxing Ren, Mingsong Li

**Affiliations:** Inflammatory Bowel Disease Research Center, Department of Gastroenterology, Guangdong Province Key Laboratory of Major Obstetric Disease, Province Clinical Research Center for Obstetrics and Gynecology, The Third Affiliated Hospital of Guangzhou Medical University, Guangzhou, Guangdong, China

**Keywords:** inflammatory bowel disease, Ustekinumab, IL12/23, Crohn’s disease, ulcerative colitis

## Abstract

Inflammatory bowel disease (IBD) is a chronic, recurrent gastrointestinal disorder with elusive etiology. Interleukin-12 (IL-12) and IL-23 have emerged as key proinflammatory mediators/cytokines in IBD pathogenesis. Ustekinumab (UST), targeting IL-12 and IL-23, has demonstrated promising efficacy and safety in the treatment of IBD. Recently, UST has become increasingly favored as a potential first-line treatment option. This review delineates UST’s mechanism of action, its clinical applications in IBD, including the response rates, strategies for dose optimization for case of partial or lost response, and potential adverse events. This review aims to offer a comprehensive understanding of UST’s role as a therapeutic option in IBD management.

## Introduction

Inflammatory bowel disease (IBD) comprises conditions like Crohn’s disease (CD), ulcerative colitis (UC), and indeterminate colitis. Characterized as a chronic and relapsing gastrointestinal tract disorder, IBD primarily manifests as inflammation, leading to a range of digestive disorders. These include abdominal pain, gastrointestinal bleeding, diarrhea, weight loss, and other debilitating symptoms, significantly impacting patients’ quality of life and escalating healthcare costs (1, 2).

The exact pathogenesis of IBD is not yet clear. Studies have identified (3) that the key factors responsible for IBD are the complex interaction among genetic components, environmental elements, dysregulated immune responses, and alterations of the microbiome. The intestinal damage caused by IBD is progressive, cumulative, and often irreversible; thus, delay in IBD diagnosis is associated with adverse outcomes; hence, early detection and prompt treatment are vital in reducing complications and improving patient prognoses (4).

Conventional treatments of IBD include aminosalicylates, corticosteroids, immunomodulators, and biologic agents (5). However, these treatments do not yield positive responses in all patients. When dietary and lifestyle modifications, conventional medication therapies, or other interventions fail to alleviate IBD symptoms, clinicians often resort to surgical options (6). Therefore, varieties of new therapeutic strategies are emerging, involving small-molecule drugs, apheresis therapy, improvement of intestinal microecology, stem cell transplantation, and exosome therapy, but these novel therapies are limited by their unclear impact on IBD and are not currently applied in clinical practice (5). Ustekinumab (UST), a human Immunoglobulin G1 (IgG1) monoclonal antibody that targets the p40 subunit of interleukin-12 (IL-12) and IL-23, has shown promise. Evidence has shown that UST is well tolerated and successful in producing and sustaining remission in patients with moderate to severe IBD who have had a clinical response to induction (7–9), with no significant side effects or adverse events (AEs) observed (10). This article aims to review the mechanism of action of UST and its application in the treatment of IBD, offering insights into its potential as an innovative therapeutic option.

## Development of UST

Because of a significant number of patients with IBD not responding adequately to conventional anti-inflammatory medications and immunomodulators, biologic agents are becoming a first-line therapy in clinical practice (11). Biologic agents mainly include tumor necrosis factor (TNF) inhibitors, anti-interleukin inhibitors, and cell adhesion molecule inhibitors. TNF inhibitors, such as infliximab and adalimumab, play an important function in IBD treatment; however, up to 40% of patients experienced primary no respond to TNF inhibitors, and 23%–46% of patients experience secondary loss of response (5), underscoring the necessity for new, effective therapeutic strategies.

Evidence from previous studies (12, 13) indicates that IL-12 plays a pivotal role in chronic intestinal inflammation, as anti–IL-12 can be effective in reversing the colitis by eliminating the T helper 1 (Th1) cells. In various inflammatory diseases, including IBD, rheumatoid arthritis, and psoriasis, IL-12 levels have been observed to increase. Oppmann et al. discovered that IL-12 p40 can change into IL-23 (p19/p40), by attaching to a protein known p19, which has different biologic functions than IL-12 (14). Similarly, IL-23 levels may also elevate in these diseases. Becker et al. identified that constitutive p40 is mainly expressed by CD8α−CD11b_CD11c^+^ lamina propria DCs in the distal ileum and elevated bacterial load in the terminal ileum fosters Nuclear Factor kappa-B (NF-κB) expression of the p40 gene; this excessive pool of p40 protein leads to high levels of IL-23, which contributes to the development of IBD (15). IL-12 and IL-23 levels are typically elevated in patients with CD and UC, serving as indicators for IBD. A genome-wide association analysis conducted by Duerr et al. (16) further demonstrated that the IL23R gene on chromosome 1p31, which encodes a subunit of the IL-23 receptor, plays a proinflammatory role in CD, suggesting that blocking the IL-23 signaling pathway is a viable therapeutic approach for IBD.

IL-12 emerged as an attractive therapeutic target for CD, leading to the development of anti-interleukin inhibitors, such as UST, which has been categorized as anti–IL-12/IL­23 antibodies following the finding of IL­23 (17). UST has been approved by the Food and Drug Administration (FDA) for the treatment of moderately to severely active IBD (11, 18). In addition, UST can be used as the first-line treatment for IBD, including for patients who have failed anti–TNF-α therapy, due to its high efficacy and safety profile (19, 20).

## Signaling pathway of IL­12 and IL­23

**Figure 1:**
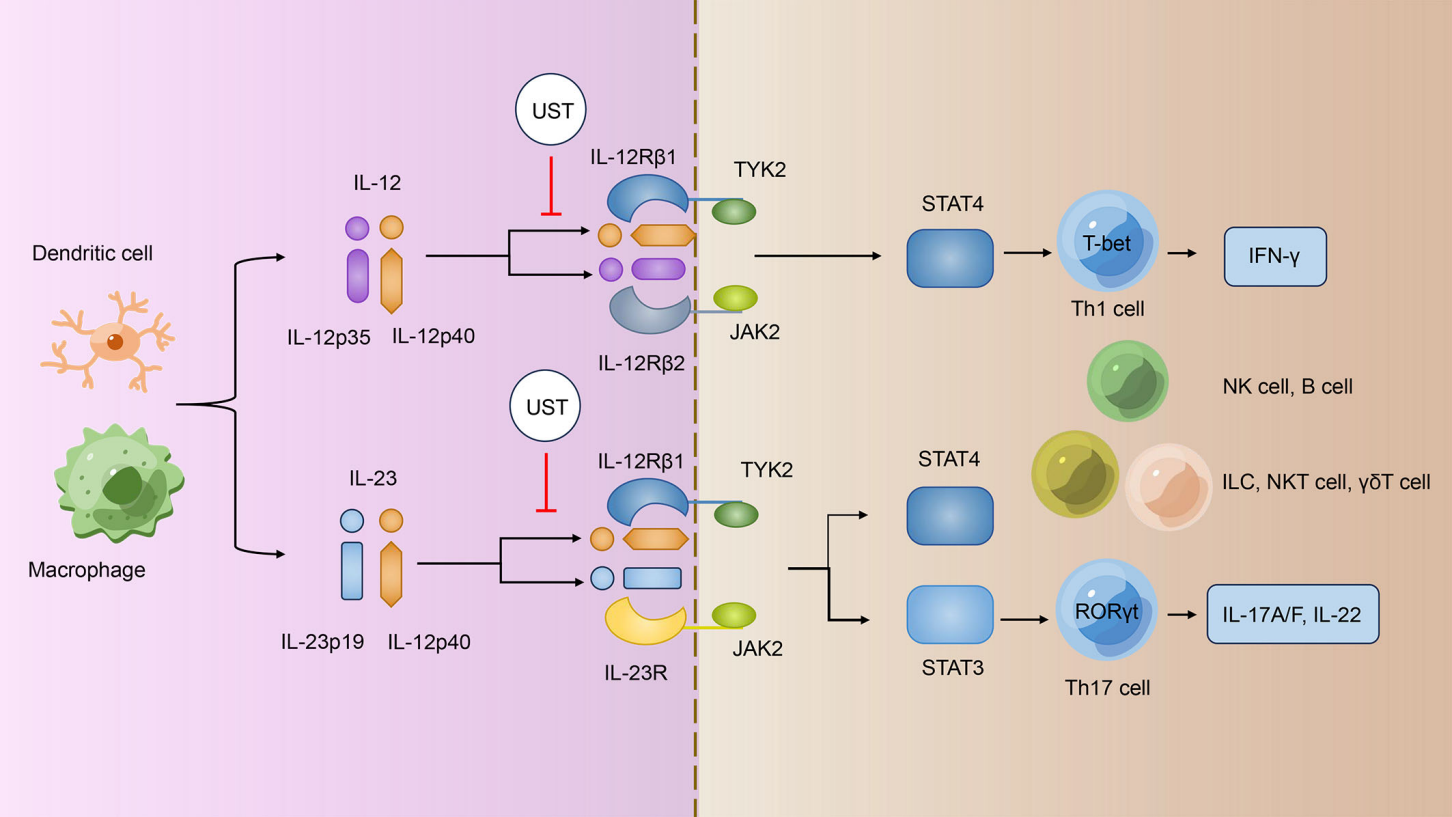
UST treatments specifically target the IL-12/IL-23 signaling pathway. IL-12 and IL-23 signal through different receptor complexes. IL-12 signals through a receptor complex composed of IL-12Rβ1 and IL-12Rβ2, whereas IL-23 signals via IL-12Rβ1 and IL-23R. Both IL­12R and IL­23R interact with the JAK family members JAK2 and TYK2 and then assist in phosphorylating of STAT4 and STAT3; IL­12 signals via pSTAT4, whereas IL­23 mainly linked to pSTAT3. The p40 subunit of both IL-12 and IL-23 attaches directly to IL-12Rβ1 and acts as a common regulator for both signaling pathways. UST targets the shared p40 subunit of IL-12 and IL-23, thereby inhibiting the development of IFN-γ–producing Th1 cells, IL-17–producing Th17 cells, and the turning on of other immune cells such as NK cells, B cells, ILCs, NKT cells, and γδT cells. UST, Ustekinumab; IL-12/23, interleukin-12/23; IL-12Rβ1/2, IL-12 receptor beta 1/2; Th1/17 cells, T helper 1/17 cells; IFN-γ, interferon-γ; NK cells, natural killer cells; ILCs, innate lymphoid cells; NKT cells, natural killer T cells.

The IL-12 family cytokines are recognized to have critical roles in the regulation of innate and adaptive immune responses, influencing the outcome of cancer, infection and inflammatory diseases (21). The IL-12 family comprises four heterodimeric cytokines: IL-12, IL-23, IL-27, and IL-35 (22). Among them, IL-12 is made up of the p40 and p35 subunits, whereas IL-23 is made up of the p40 and p19 subunits; their corresponding cytokine receptors are also heterodimeric, and both IL­12 and IL­23 cytokine receptors share the IL-12 receptor beta 1 (IL-12Rβ1) subunit (22). IL-12 signals through a receptor complex composed of IL-12Rβ1 and IL-12Rβ2, whereas IL-23 signals via IL-12Rβ1 and IL-23 receptor (IL-23R) (**Figure 1**) (22). The provided p40 subunit of IL-12 and IL-23 binds directly to IL-12Rβ1 and acts as a shared regulator of IL-12 and IL-23 communicating, and the functional distinctions between IL-12 and IL-23 are possibly due to their distinct four-helix bundle components, IL-12p35 and IL-23p19, which bind particularly to IL-12Rβ2 and IL-23R, respectively (23).

Janus kinase–signal regulators and triggers of transcription (JAK-STAT) family are operational by tyrosine phosphorylation in response to IL-12 family and mediate their signaling (24). Both IL­12R and IL­23R connect with the JAK family members JAK2 and TYK2 and then promote the phosphorylation of STAT4 and STAT3; IL­12 signals via pSTAT4 (25, 26), whereas IL­23 most prominently linked to pSTAT3 (**Figure 1**) (27). Subsequently, pSTAT3 and pSTAT4 bind to their target genes and regulates gene expression.

## Biological functions of IL-12 and IL-23

IL-12 can be produced by antigen-presenting cells including dendritic cells (DCs), macrophages, granulocytes, and B cells (28, 29), whereas IL-23 is produced by activated DCs and macrophages (30), and recent study also found that neutrophils can secrete IL-23 (31).

IL-12 is essential to the functions of T cells and natural killer (NK) cells (29). It was early identified that STAT4 targets and activates interferon-γ (IFN-γ) gene in T cells and NK cells in response to IL-12 (32). Research also demonstrated that STAT4-deficient mice showed impaired IL-12–mediated Th1 differentiation, IFN-γ induction, and impaired NK cytotoxicity (25, 33). However, IL-12 is not sufficient to induce development of naive CD4^+^T cells to Th1 cells as IL-12Rβ2 is not expressed by naive resting CD4^+^ T cells (34). T-bet is induced by IFN-γ–STAT1 signaling during T-cell activation, which can rapidly upregulated IL-12Rβ2 chain expression (35, 36). When IFN-γ is induced by the IL-12 signaling, the upregulation of T-bet is further intensified, forming a positive feedback loop. IL-12 can also drive B cell secrete immunoglobulin isotypes associate with Th1 response indirectly (37).

IL-23 does not stimulate the growth of IFN-γ–producing Th1 cells; instead, it promotes the differentiation of Th17 cells and maintains the expression of IL-17 (38). Similarly, IL­23 alone is not sufficient to trigger Th17 cell development due to the absence of the IL­23R on naive T cell (39, 40). Ivanov et al. found that IL-6 and transforming growth factor–β produced by activated DCs and other cells can activate the expression of retinoid­related orphan receptor–γt (RORγt), which is a key regulator that promotes the expression of IL­17 and can upregulate IL-23R in RORγt^+^ T cells (40). IL-23R signaling subsequently activates STAT3, which maintains RORγt expression and, in turn, promotes the transcription of IL­23R, building up a positive feedback loop, and enhances IL-17 gene transcription (41, 42).In addition to Th17 cells, natural immune cells that express RORγt, such as subsets of γδT cells, NK T (NKT) cells, “natural” Th17 cells, and innate lymphoid cells (ILCs), also respond to IL-23 and have an essential role in both resisting infection and mediating autoimmune pathology (41). Finally, both IL-12 and IL-23 are integral to maintaining the integrity and barrier function of the intestinal mucosa, preventing gut bacteria and toxins from entering the bloodstream. IL-12 enhances tight junction protein expression and distribution, bolstering the intestinal mucosa’s barrier function. IL-23 participates in inflammatory and immune responses, promoting the proliferation and repair of intestinal mucosal epithelial cells and maintaining the mucosa’s integrity and barrier function. Moreover, IL-23 plays a role in maintaining the balance of the intestinal microecology, further protecting the mucosal barrier (42, 43).

## Anti–IL-12/IL-23 inhibitors

Although anti-TNF agents are the primary therapeutic option for patients with IBD with complications, nearly two-thirds of patients experience either a primary nonresponse or a secondary loss of response to anti-TNF agents, and the risks of infection and malignancy of anti-TNF agents should be considered (44). Consequently, anti–IL-12/IL-23 inhibitors, such as UST, Risankizumab, Brazikumab, Mirikizumab, and Guselkumab, were developed as the new alternative therapies for clinical management of IBD. Among them, UST has been approved for clinical usage in individuals with IBD, and Risankizumab has been recently approved for the treatment of moderate-to-severe active CD; the clinical remission rate in patients with moderate to severe CD treated with Risankizumab is 40%–45%, with an endoscopic remission rate of 29%–40% (45), whereas the remaining IL-23–specific inhibitors are currently undergoing tested at different stages (46, 47). It is clear that IL-12/23 inhibitors are an indispensable treatment option for IBD, with UST currently showing the best efficacy and safety. These novel IL-23–specific inhibitors currently being developed can function without restriction of IL-12–dependent T-cell pathway, resulting in safer therapy due to its protection against infection and malignancy (46, 47). Because of the role of IL-12 in driving colon inflammation during the early stages of IBD and the subsequent shift toward an IL-23–dependent inflammatory response contributing to disease chronicity (48), it is indicated that anti–IL-23p19 antibodies may be more effective in the later stages of IBD (49). Both the safety and effectiveness profile of IL-23–specific inhibitors still need to be further demonstrated in phase 2 and phase 3 studies.

UST is a human IgG1 monoclonal antibody that recognizes the p40 subunit shared by IL-12 and IL-23; real-world evidence has shown that UST is successful in inducing and maintaining remission in patients with IBD who are refractory to anti-TNF agents or conventional therapy (7, 8). In addition, UST was demonstrated to be effective in perianal refractory CD and other extra-intestinal manifestations (50, 51) and is also associated with a lower risk of infection or other AEs (44, 52, 53). Therefore, among elderly individuals who are at a higher risk of infection or have recently undergone cancer treatment, UST may be a more favorable option compared to anti-TNF therapy. Moreover, previous report has shown that it not only has systemic anti-inflammatory effect but also lacks immunogenicity (54), suggesting that concurrent immunosuppression is unnecessary and the safety is greater than anti-TNF agents.

## Clinical applications of UST

### CD

The UNITI-1/2 and IM-UNITI studies (Clinical Trial Registration: NCT01369329, NCT01369342, NCT01369355) demonstrated that patients with CD receiving intravenous UST at a dose of either 130 mg or about 6 mg/kg responded much better than those receiving placebo. In UNITI-1/2 trials, the response rates at week 6 exceeded one-third and one-half, respectively. Similarly, IM-UNITI trial also showed a response rate of approximately half at week 44 (7). A 252-week long-term extension (LTE) study examined the long-term efficacy, safety, and immunogenicity of subcutaneous (SC) UST maintenance therapy in patients with CD and found that 34.4% of patients in the q8w group and 28.7% in the q12w group were in clinical remission at the study end point. This study demonstrated that long-term SC UST therapy was well tolerated and successful to keep clinical remission for nearly 5 years in TNF antagonist-naive patients (52). In addition, study also found that up to 77.4% of patients treated with UST achieved fistula response at week 252, and no evidence showed that UST increase the incidence of malignancy, anaphylactic shock, or delayed reactions due to hypersensitivity (52). A prospective, head‐to‐head trial has shown that, in patients with CD with prior failure to anti‐TNF treatment, UST is more effective in achieving corticosteroid-free clinical remission and/or biochemical remission compared to vedolizumab (55). A phase 3b trial conducted by Sands et al. has recently shown that, in biologic-naive patients with moderately to severely active CD, Adalimumab and UST given as single-therapy were extremely effective for a full year without continuous immunosuppression. However, there was no evidence that UST was superior to adalimumab in terms of clinical remission at week 52, and the immunogenicity and safety results were consistent with the known characteristics of both biologic drugs (56). Moreover, a real-world study reveals that bio-naive patients who undergo UST treatment can achieve higher rates of improvement compared to bio-experienced patients (57). The latest research published confirms that flexible UST maintenance dosing can maintain clinical and endoscopic efficacy in patients with CD up to 104 weeks. The safety and tolerability are good with low rates of serious AEs (SAEs) (58).

### UC

Although the impact of UST on UC is not as significant as that on CD, it still plays a crucial role in inducing and maintaining long-term remission of UC symptoms. Sands et al. (8) have shown that, in patients with moderate to severe UC treated with UST, the clinical remission rates were significantly higher than those who received placebo. Approximately 15% patient using UST as induction therapy achieve clinical remission at week 8, and the percentage of patients who achieved clinical remission at week 44 with maintenance therapy every 8 weeks and every 12 weeks was 43.8% and 38.4%, respectively. The incidence of SAEs was comparable to that of placebo. Another study (59) also indicated that the induction and maintenance therapy of UST result in significantly higher rates of histologic improvement at week 8 and week 44 compared to placebo. Up to 61% of patients with UC treated with UST were able to achieve histo-endoscopic mucosal healing at week 44. The UNIFI LTE study (60) has reported that, in patients with UC who received UST treatment q12w and q8w, the corticosteroid-free symptomatic remission rate at week 152 was 51.2% and 55.1%, respectively. Nasopharyngitis and upper respiratory tract infection were the most commonly reported AEs and UC worsening. The AE and SAE rates of UST treatment at week 156 were comparable to those of the placebo group. This study confirms that UST is effective in maintained symptomatic remission in patients with moderately to severely active UC through the third year of maintenance treatment. In addition, long-term UST maintenance therapy has been shown to be effective in patients with UC through 4 years of follow-up. In a 348-patient study, 55.2% achieved symptom relief, and approximately 80% showed endoscopic improvement when reviewing 171 patients. The long-term efficacy of UST maintenance in patients with UC has been confirmed through 4 years of follow-up, with no new safety signals observed (61).

### Dosage

UST should be administered intravenously at a dosage of approximately 6 mg/kg (260 mg under 55 kg; 390 mg between 55 kg and 85 kg; and 520 mg over 85 kg) at week 0; patients who respond to intravenous (IV) induction subsequently receive 90 mg subcutaneously every 8 weeks or every 12 weeks. Because of the pharmacokinetic features of UST and relationship between exposure and response, it has been shown that there was a substantial difference in clinical remission rate between the two groups. Patients who received SC UST maintenance therapy every 12 weeks experienced a lower remission rate than those who received treatments every 8 weeks (52, 56) because the trough concentration of UST in patients who receive maintenance therapy every 8 weeks was three times higher than those who receive it every 12 weeks (62). In addition, it has been confirmed that the combination of immunosuppressants did not have significant impact on the serum concentration of UST, and the trough concentration of UST of 0.8–1.4 mg/mL was associated with maintenance of clinical and endoscopic remission (62). However, the optimal cutoff value of UST trough concentration is still debated.

### Safety profile

**Figure 2:**
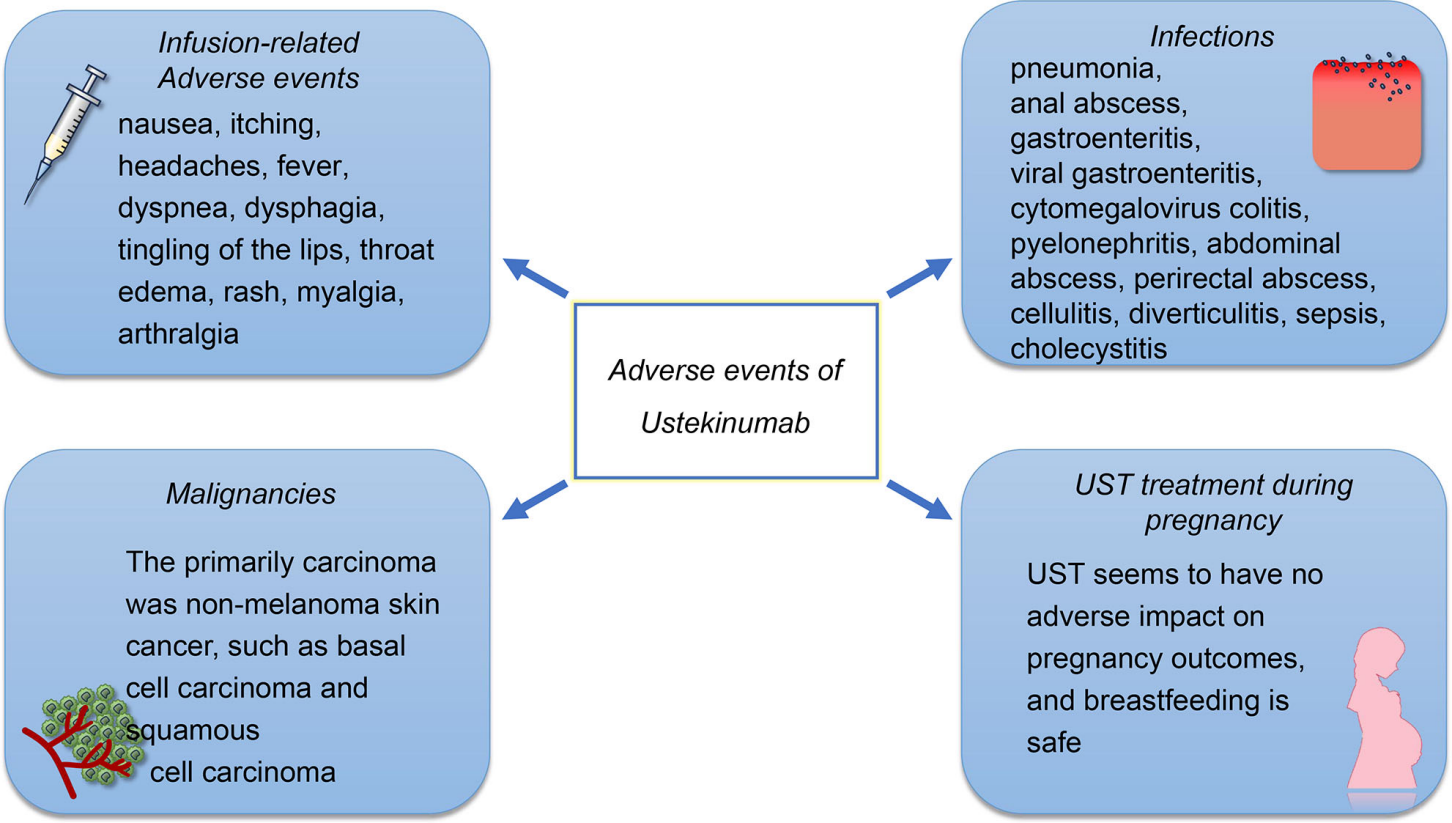
The safety profile of UST. The AEs of UST treatment include infusion reactions, infections, and malignancies. The infusion-related adverse events were rarely reported, in which 1% of patients treated with UST occurred injection-site reactions and 2% of patients experienced infusion-related AEs. The most frequently reported infections of UST treatment are pulmonary and gastrointestinal. However, the infection rates for patients who received UST treatment is low compared to those who received other biological agents. Although the elderly experience a higher incidence of carcinoma, the incidence of malignancies such as basal cell carcinoma and squamous cell carcinoma is also low. UST appears to be safe on the development of the immune system and produced no AEs on the pregnant women and infants.

The reported AEs include infusion reactions, infections, malignancies, and gastrointestinal disorders. Pregnancy-related AEs should also be a concern, as the active disease during pregnancy may increase the risk of adverse birth outcomes (**Figure 2**). However, there is limited evidence on the safety of UST during pregnancy. The frequency of adverse drug reactions (ADRs) and serious ADRs (SADRs) was generally low in patients treated with UST. The post‐marketing surveillance in Japan (63) shown that the overall incidences of ADRs and SADRs were 5.3% and 2.1%, respectively. A study in Brazil shown that, although most of patients might experience at least one AE, most of the AEs were mild or moderate and were not connected to the UST, with the SAE rate being 21% and with the ADR rate being 44% (64). A prospective cohort study conducted in The Netherlands (65) revealed that the ADRs associated with UST were 13.4 per 100 patient-years, and only 7% of patients discontinued UST due to ADRs. Furthermore, the study found that the overall rate of ADRs in the second year of treatment was lower compared to that in the first year. These findings suggest that the majority of severe ADRs occur during the early stages of UST treatment. Future research is necessary to enhance our comprehension of the pathophysiology that underlies these AEs.

### Infusion-related AEs

Acute infusion reactions include nausea, itching, headaches, and fever. Infusion reactions can be prevented by slowing down the rate of infusion, taking antihistamines, and administering intravenous glucocorticoids before the infusion. In case of significant acute infusion reactions, glucocorticoids can be administered to alleviate these symptoms. However, only 1% of patients treated with UST occurred injection-site reactions and 2% of patients experienced infusion-related AEs (56). A delayed infusion reaction may occur within 2 weeks of the infusion. The cause of this reaction is a type III hypersensitivity reaction, which presents as a rash, myalgia, arthralgia, headache, and fever. The pathophysiology underlying infusion reactions to UST remains unknown.

### Infections

UST is effective in reducing inflammation; however, it also has an impact on the immune system, as IL12/23 signaling pathway plays an essential role in resisting infection. The infections that are reported most often are pulmonary and gastrointestinal (66). Serious infections at a rate of ≥0.1 included pneumonia, anal abscess, gastroenteritis, viral gastroenteritis, and cytomegalovirus colitis (10, 52). Other pathogen infections that have been reported included active mycobacterium tuberculosis, esophageal candidiasis, legionella pneumonia, and concurrent ophthalmic and oral herpes simplex (10, 67). The data have shown that UST could potentially offer a greater net benefit to patients with CD, compared to TNF-α antagonists and vedolizumab, owing to its lower risk of serious infections (68).

### Malignancies

The incidence of cancer among patients who receive UST was low, and there was no significant difference between the UST patients and the placebo group. The primarily carcinoma was non-melanoma skin cancer (NMSC), such as basal cell carcinoma and squamous cell carcinoma (67). In a different study, it was found that none of the 100 patients with IBD had lymphoma. However, four cases of basal cell carcinoma and three cases of squamous cell carcinoma were observed (10). Apart from NMSC, the incidence of other malignancies is low, and the incidence among placebo and UST patients is similar (10). There is still limited evidence for interpreting the incidence of malignancy, and further investigations are required.

### UST treatment during pregnancy

The IL-12/23 signaling pathway also associated with the damage during embryo implantation and subsequent trophoblast development (69). In a study conducted on animals, it was determined that UST was safe on the development of the immune system and produced no adverse effects on the pregnant women and infants (70). Based on the limited data in case reports, it is unlikely that UST will have a negative impact on pregnancy outcomes, and breastfeeding is pointed out to be safe as the concentration of UST in breast milk is low (71, 72). Despite the study also discovering that the breast milk levels after re-introduction of treatment were similar to the serum trough levels, the pregnancy outcome was successful and no birth defects were observed (73). In summary, UST appears to be safe during pregnancy, although more studies are needed to confirm this.

## Conclusion

Ustekinumab (UST), as an FDA-approved biological therapy, has demonstrated its efficacy in both inducing and sustaining remission in diseases by targeting the IL-12/23 pathway. Primarily utilized in the treatment of psoriasis and IBD, UST has shown notable success, especially in managing CD. It also effectively maintains remission in UC, expanding its therapeutic scope. In the realm of IL-23–specific inhibitors, Mirikizumab, for instance, has exhibited promising results in treating patients with UC (49). These novel inhibitors, focusing specifically on IL-23, are considered to be safer therapies, although they are still under rigorous evaluation in various phases of randomized controlled trials.

Although UST is often regarded as a secondary option for patients who do not respond to primary anti-TNF therapy, it is important to recognize the potential for partial or complete loss of response to UST as well. This necessitates strategies like dose escalation or intravenous reinduction to boost efficacy, fortunately without significantly increasing the risk of AEs. Despite UST’s high safety profile and fewer side effects compared to other biologics—a benefit attributed to its human monoclonal nature—there remains a risk for some patients to develop acute allergic reactions or infections. Given that biological therapies can substantially modify the local immune response, it is crucial to understand and assess the individual immune environment. This approach aids in predicting therapeutic responses and tailoring treatment strategies, emphasizing the importance of personalized medicine in IBD management (74). In conclusion, UST emerges as a robust, effective, and safe treatment option for patients with IBD, balancing its advantages against any potential drawbacks. UST has shown good therapeutic effects in clinical practice and can be used as a first-line medication. Further research is needed to determine whether UST, as a first-time biological agent, will have better therapeutic effects. Its role in managing IBD highlights the continuous evolution and refinement of treatment modalities in the field.

## Author contributions

WZ: Writing – original draft. GZ: Writing – original draft. XR: Writing – review & editing. ML: Writing – review & editing.
